# Stimulatory Thyrotropin Receptor Antibodies Are a Biomarker for Graves’ Orbitopathy

**DOI:** 10.3389/fendo.2020.629925

**Published:** 2021-02-02

**Authors:** Augustine George, Tanja Diana, Jan Längericht, George J. Kahaly

**Affiliations:** Molecular Thyroid Research Laboratory, Department of Medicine I, Johannes Gutenberg University (JGU) Medical Center, Mainz, Germany

**Keywords:** functional thyrotropin receptor antibodies, stimulatory TSH receptor antibodies, Graves’ orbitopathy, thyroid eye disease, biomarker

## Introduction

Graves’ Orbitopathy (GO) or thyroid eye disease (TED) is the most common and prominent extrathyroidal manifestation in patients diagnosed with Graves’ disease (GD). Approximately 50% of patients diagnosed with GD develop GO, and with further orbital imaging, up to 80% of patients test positive for TED (1). Physical signs and symptoms (2), as well as the impact on the psychological well-being (3, 4) and quality of life (5, 6), underline the importance of effective disease management. Furthermore, the incidence of TED is linked to autoimmune gastritis and coeliac disease (7), implicating the importance of diagnosis of GO in patients, to identify risk factors for gastrointestinal autoimmunity.

Current evidence indicates that stimulating thyrotropin receptor IgG antibodies (stim. TSH-R-Ab or TSAb) are the main causative agent of GO, making its measurement a useful tool to predict and assess both clinical disease severity and activity.

## Terminology

Numerous terms have been established to describe TSH-R-Ab, the different nomenclature refers to various types of immunoassays (8). Total TSH-R-Ab also referred to as TRAb, are Ab that interact distinctively with the TSH-R. Routinely, these Ab are gauged by competitive immune binding assays and therefore are termed TSH-R-binding inhibitory immunoglobulins (TBII). Since binding assays only assess the binding of Ab to the TSH-R, it cannot display the function of the Ab it tests. Cell-based bioassays however differentiate between Ab that block or stimulate the TSH-R. Stimulating Ab are termed thyroid-stimulating Ab (TSAb) or thyroid-stimulating immunoglobulins (TSI), while blocking Ab are referred to as thyroid blocking Ab (TBAb) or thyroid blocking immunoglobulins (TBI). Further alternative terms for TBAb are TSH-R-stimulating-blocking Ab and TSH-R-blocking Ab or TRBAb.

## Distinction Between Blocking and Stimulating Antibodies

TSH-R-Ab are found in patients with autoimmune thyroid disease (AITD) and play a major role in their pathogenesis and clinical presentation (9). TSH-R-Ab are divided into three groups since they have different ways to interact with the TSH-R. TSAb bind to the large extracellular amino-terminal and cause a stimulation of the TSH-R. This results in increased cyclic adenosine 3’, 5’-monophosphate (cAMP), which increases the transcription of proteins necessary for the synthesis of thyroxine (T4), and triiodothyronine (T3). The outcome of this pathway is higher synthesis rates of thyroid-related hormones T3 and T4, and increased proliferation of the thyroid follicular endothelial cell. On the other hand, TBAb inhibit the function of the TSH-R, decreasing the synthesis rate of thyroid hormones, and inhibiting the proliferation and growth of the thyroid cell (10, 11). Other TSH-R-Ab neither block nor stimulate but rather have a neutral effect on the TSH-R. These Ab are referred to as neutral Ab or “cleavage” Ab and are not fully understood in their respective effect *in vivo*. Neutral Ab are able to induce various signaling cascades including some of which are initiated by TSAb, as well. However, neutral Ab can induce unique downstream signaling cascades including the activation of protein kinase C/mitogen activated protein kinase (MAPK), mammalian target of rapamycin (mTOR), nuclear factor ‘kappa light chain enhancer’ of activated B-cells (NF-κB), different cytokines, and reactive oxygen species (ROS). The initiation of these pathways is yet unclear, a recruitment of multiple G-proteins is possible. *In vitro* experiments show the expression of heat shock Proteins (p27, p107), endoplasmic reticulum stress protein (grp98), various oncogenes (p53, p73, retinoblastoma protein), and apoptosis of rat thyrocytes after a period of exposure to neutral Ab. It is unclear if ROS alone, or other signaling cascades play a role in the initiation of apoptosis (12, 13).

## Role of Functional TSH-R Autoantibodies in Go

**Figure 1:**
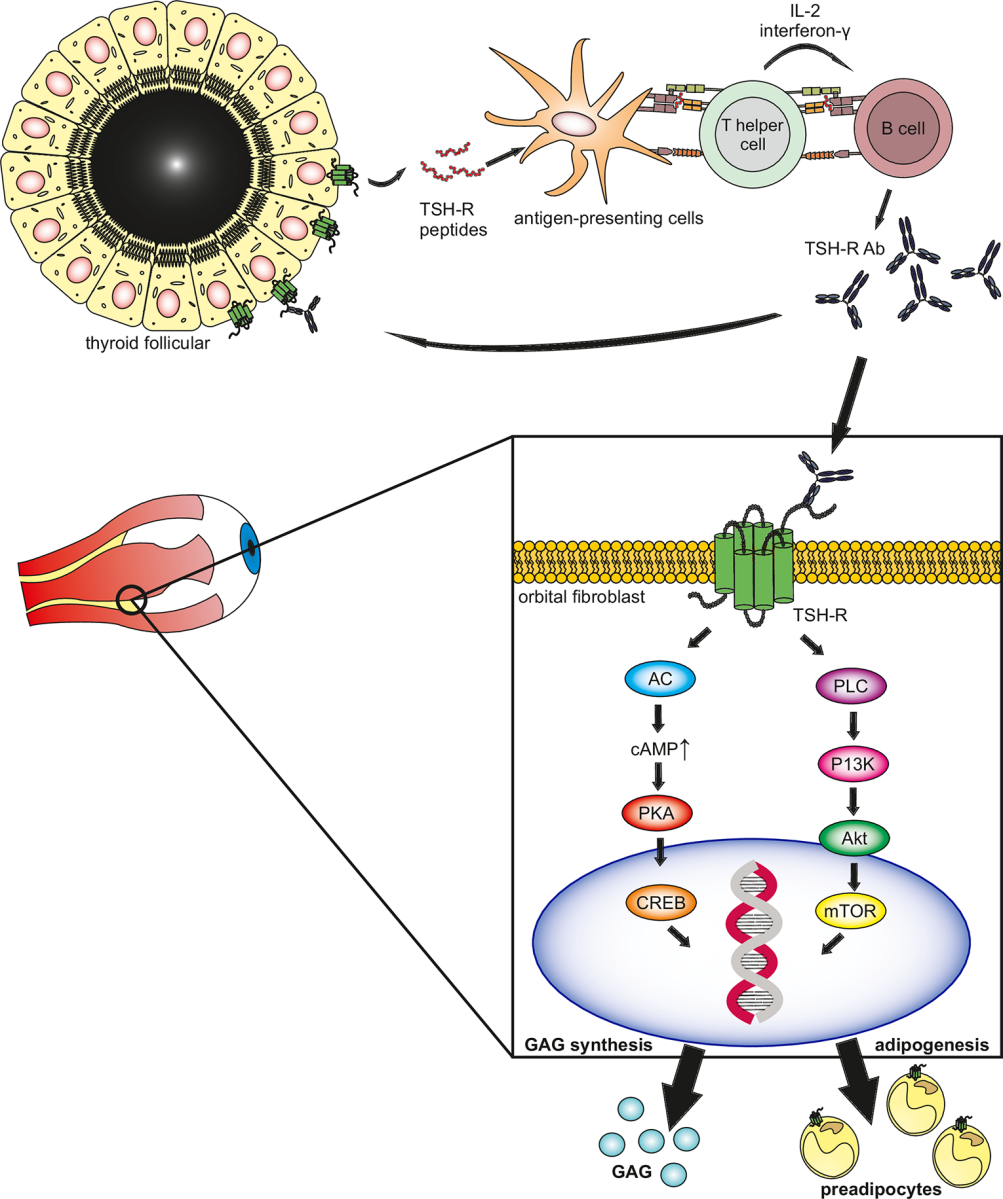
The thyrotropin receptor (TSH-R) is the main autoantigen in Graves’ hyperthyroidism and associated eye disease. TSH-R peptides are ingested by antigen-presenting cells (APC) through phagocytosis and expressed through MHC class II. T-helper cells recognize the antigen, by binding with the T-cell receptor, along with CD154 to its ligand CD40 on the surface of the APC. The activated T-helper cells bind to B-cells, transforming them into TSH-R antibody-secreting plasma cells through inflammatory cytokines interleukin II and gamma interferon. The synthesized TSAb bind to the TSH-R expressed by thyrocytes and orbital target cells (fibroblasts, pre-adipocytes), activating the Gαs adenylyl cyclase (AC) pathway. This stimulates protein kinase A which induces gene activation through the cAMP responsive element binding (CREB) protein. Additionally, the Gαq protein kinase C (PKC) pathway activates protein kinase B (Akt) inducing the mammalian target of rapamycin (mTOR) further inducing gene expression. The induction of gene expression lead to differentiation into pre-adipocytes and synthesis of hydrophilic mucopolysaccharides (glycosaminoglycans), hence leading to edema and later fibrosis in the orbital space, resulting in the clinical phenotype of thyroid eye disease.

GO/TED is characterized by a protrusion of the eyes, upper lid retraction, diplopia, and irritation of the periorbital tissue and conjunctiva. In a study including 101 consecutive patients with TED, none tested positive for TBAb while 91 (90%) showed presence of TSAb of whom 90 were diagnosed with GD (14), concluding that not TBAb but rather TSAb are greatly prevalent in patients with TED. Current evidence suggests that TED is primarily caused by TSAb, which are present in patients with GD and Hashimoto’s thyroiditis (HT). Animal models support the hypothesis of TSAb and TBAb, being synthesized as a result of immunization with the TSH-R, detected as an auto-antigen (15). The TSH-R is physiologically expressed in the soft tissue of the orbit, which was demonstrated in experiments where TSH-R mRNA was detected in affected tissue of the eye socket (16). Another study independently noted an overexpression of the TSH-R and human leukocyte antigen-DR (HLA-DR) in patients with GO (17). Produced by B- cells, TSAb enter the bloodstream and stimulate the TSH-R in various tissues e.g., the thyroid gland, orbital soft tissue, skin, and heart. In orbital fibroblasts, the Ab induce differentiation into pre-adipocytes and secretion of hydrophilic glycosaminoglycans (GAG), resulting in edema and later fibrosis (18, 19) (**Figure 1**).

Inflammatory cytokines play a major role in the pathogenesis of GO. By binding to the TSH-R, TSAb induces inflammation through inflammatory cytokines such as tumor necrosis factor-α (TNF-α), interferon-γ, and interleukin-1 which perpetuate the synthesis of collagen, GAG, and differentiation of orbital fibroblasts into adipocytes (20). Furthermore, by inducing the expression of heat shock proteins, prostaglandins, adhesion molecules, CD40, and major histocompatibility complex protein class two, cytokines amplify the inflammation cascade (21). The importance of inflammation explains the success of anti-inflammatory drugs like mycophenolate and glucocorticoids in the treatment of GO (22–24). The pathogenesis of GO is further supported by evidence, as high TSAb titers are associated with GO in patients with GD (25).

## Clinical Relevance of TSAB as a Biomarker For Go

Since the clinical presentation of GD and GO, especially the eye protrusion, is caused by stimulating TSH-R-Ab, measurement of TSAb is an excellent tool to manage patients with GD/GO. As there is a need to assess the function of the Ab, a cell-based bioassay rather than a binding assay is preferred (26, 27). In cell-based bioassays, IgG Ab are gauged through the signal transduction of cAMP, mediated through genetically engineered TSH-R on intact cells. Previously, the concentration of cAMP was measured through radiolabeled immunoassays (RIA). More recently, the amount of cAMP is assessed with cAMP-inducible reporter genes that express the luciferase enzyme. The enzyme concentration is then gauged with a luminometer after the luciferase substrate is added. TBAb are measured in an analogous bioassay while measuring their ability to competitively block the TSH-R against bovine TSH. In contrast, clinically employed immune binding assays assess the amount of TRAb, by measuring the displacement of a tracer, which is either radioiodine labeled bovine TSH or a monoclonal mouse antibody (MAb) with affinity to the TSH-R.

Previously, cell-based bioassays were toilsome and complicated procedures with varying specificity and sensitivity between laboratories, making it an unreliable way to measure Ab in comparison to binding assays. However, through major improvements in genetic engineering and molecular cloning, cell-based bioassays have become much more reliable and easier to perform. To improve reproducibility, the use of transfected cell lines of the Chinese ovarian hamster (CHO) was established, making the procedure more convenient with an improvement of assay sensitivity and specificity. Even though binding TSH-R-Ab or TBII can be used for differential diagnosis of GO (28), cell-based bioassays significantly outperform binding assays in terms of sensitivity and accuracy (27, 29–31), making it a better choice for management of GO.

Several studies were conducted to assess the reliability of TSAb as predictor for GO. A report investigating the relation of TSH-R-Ab and GO underlines the benefits of using functional Ab in the clinical routine (32). A total of 155 GD patients were tested for TSAb, using two different types of cell lines. All hyperthyroid patients with GD and TED tested positive for TSAb/TSI and TSAb were detected in 150 of 155 patients with TED. None of the 40 controls was Ab positive. TSAb levels in patients with GO were 3-fold and 8-fold higher than in GD patients and healthy controls, respectively.

To evaluate the relation between TSAb and the clinical presentation of TED, two standardized classifications were utilized. A clinical activity score (CAS), determined through an author unaware of the measured lab data, was used. To gauge the severity of TED, a clinical severity score (CSS) was utilized, based on the NOSPECS classification (33). TSAb titer correlated with both severity (r = 0.87, p < 0.001) and activity (r = 0.87, p < 0.001). In contrast, radiolabeled assays (RIA) showed weaker correlations at r = 0.17, p < 0.015 and r = 0.54, p < 0.001, respectively. Further, a cross-sectional trial assessing the clinical relevance of TSI in regards to TED, showed a similar correlation between severity (r = 0.83, p < 0.001) and activity (r = 0.81, p < 0.001) (34). A more significant association of TSAb with clinical features of GO was observed than TBII and thus TSAb may be regarded as functional biomarker for GO.

A multicenter cross-sectional study evaluated the relation of TSAb and TED in children with GD (35). 422 samples from 157 children with GD, 101 samples from non-thyroidal autoimmune diseases, and 50 healthy controls were tested. All patients with GD+GO tested positive for TSAb, compared to 96% in the binding assay (both p < 0.001). Further, in euthyroid children with GO, TBII were positive in 24 of 31 (77%) children only, while TSI were detected in all subjects (both p = 0.016). Additionally, in a different trial (36), children with GD+GO displayed the highest titers of TSAb (SRR% 417±135) in comparison to GD only (SRR% 320±157). Additionally, hyperthyroid children with GD+GO displayed higher TSI levels compared to children with GD only (median SRR%, 481 vs. 395%, both p < 0.001). TBII however, failed to differ between the two groups (p < 0.125). With the classification of disease severity, children were distinguished in moderate-to-severe and mild GO according to the classification of the European Group on Graves’ Orbitopathy (EUGOGO). As in adults, children with moderate-to-severe GO exhibited higher TSAb titers compared to cases with mild GO (median SRR% 536 vs. 259, p < 0.001). Following the subjects during an average 3-year antithyroid drug (ATD) treatment, TSAb decreased by 69% and 20% in patients with GD and GD+GO, respectively. In contrast, TBII titers did not significantly differ between the two groups (90 vs. 89% in GD vs. GD+GO). Hence, TSAb is a strong indicator and predictor of GO in children (37), and supports the use of TSI/TSAb for management of pediatric GD/GO.

Furthermore, a 2-year prospective trial was conducted to evaluate the success of ATD treatment of one hundred consecutive hyperthyroid adults with GD, by measuring functional Ab and TBII (38). Forty-four of one hundred patients responded to ATD with Methimazole (MMI), of whom 43% suffered from GO. In the group with 56 non-responders, 66% of patients were diagnosed with GO. TSAb mirrored the activity of GD and was able to differentiate responders from non-responders. Additionally, and in contrast to TBII, TSAb titers were higher in adults with GO+GD, compared to GD alone. Finally, studies from Japan and Singapore also support the fact that TSAb correlate with the severity of GO. Indeed, increased TSAb titers are found in adults with higher GO scores (39), and in patients with GD+GO vs. GD only (40). As shown previously, GO activity did not correlate with serum TBII levels.

## Clinical Utility of Functional TSH-R-Ab

Cell-based bioassays exclusively and solely differentiate between blocking and stimulating TSH-R-Ab and can measure TSH-R-Ab at very low concentrations (27). The presence of TSI/TSAb is predictive for GO (39). In contrast, TBII is not associated with GO. The utilization of TSAb facilitates rapid diagnosis of GD and allows differential diagnosis of thyrotoxicosis, being the only biomarker able to reliably differentiate GO+GD from patients with GD only. In the clinical routine, using early *in vitro* testing of TSAb showed a 46% faster time to diagnosis of GD and 47% cost savings since costly procedures and consultation of specialists was reduced (41). During specific treatment, TSAb detects patients with GO and can predict their responsiveness to therapy. Therefore, TSAb is the superior tool to manage GO in pediatric and adult patients (9, 20, 37, 42). Recently, an immunoassay has been developed to measure TSH-R-Ab using the bridge technology (43). It has been assumed that this assay detects TSAb only. The assay utilizes a pair of recombinant TSH-R and TSH-R-Ab are measured by binding one antibody arm to a capture receptor on the solid phase and bridging with the other arm to a detection receptor generating a signal. However, numerous comparative studies have proved that the bridge immunoassay is not able to differentiate between functional TSH-R-Ab (stimulating or blocking). Indeed, twenty hypothyroid Hashimoto’s thyroiditis (HT) patients with high titers of TBAb measured in a blocking TSH-R-Ab cell-based bioassay, tested all positive in the bridge immunoassay (30). In another study, ten samples from TBAb-positive/TSAb-negative (both measured in bioassays) patients with GD or HT were positive in the bridge immunoassay (31). Further, various mixtures of monoclonal antibodies (MAb) of M22 and K1-70 were positive detected in the bridge assay. A recent study revealed that TBAb were present in one patient with HT and in two patients with GD and these patients were also positive in the bridge immunoassay but negative in the TSAb bioassay (29). Furthermore, all TBAb positive samples in a Graves’ disease animal model were positive in the bridge assay without exception (15). Therefore, the bridge assay is a purely binding immunoassay. In conclusion, although not standardized yet and requiring more time and experienced lab personal, functional TSH-R-Ab in general and stimulatory Ab in particular are clinically useful and have been demonstrated to be a reliable and accurate biomarker for the diagnosis, differential diagnosis and monitoring of patients with GO.

## Author Contributions

Literature search, writing, text revision, layout: AG. Primary concept, writing, editing, critical evaluation, supervision: GK. Critical evaluation and editorial assistance: TD and JL. All authors contributed to the article and approved the submitted version.

## Conflict of Interest

GK consults for Immunovant, Mediomics, Merck, Novartis, and Quidel.

The remaining authors declare that the research was conducted in the absence of any commercial or financial relationships that could be construed as a potential conflict of interest.

The JGU Medical Center has received research-associated funding unrelated to this study from the JGU Medical Faculty, AdvanceCor, Germany, Apitope, United Kingdom; Immunovant, USA, ISAR, Germany, Horizon, USA, Mediomics, USA, Merck, Germany, Novartis, USA, Quidel, USA, River Vision, USA, and Roche, Switzerland.

This article was not specifically funded by a company. The funder was not involved in the study design, collection, analysis, interpretation of data, the writing of this article, or the decision to submit it for publication.
